# Scabies Diagnosis: A Narrative Review of Diagnostic Methods, Clinical Misdiagnosis, and Emerging Directions

**DOI:** 10.3390/jcm15197346

**Published:** 2026-09-22

**Authors:** Saptorshi Gupta, Simon Thornley, Cameron Grant, Arthur Morris, Gerhard Sundborn

**Affiliations:** 1 Section of Epidemiology and Biostatistics, Faculty of Medical and Health Sciences, The University of Auckland, Auckland 1010, New Zealand; s.thornley@auckland.ac.nz; 2 Department of Paediatrics, Child and Youth Health, Faculty of Medical and Health Sciences, The University of Auckland, Auckland 1010, New Zealand; cc.grant@auckland.ac.nz; 3 LabPlus, Auckland 1148, New Zealand; arthur.morris@tewhatuora.govt.nz; 4 Department of Pacific Health, Faculty of Medical and Health Sciences, The University of Auckland, Auckland 1010, New Zealand; g.sundborn@auckland.ac.nz

**Keywords:** scabies, diagnosis, diagnostic accuracy, IACS

## Abstract

Caused by the mite *Sarcoptes scabiei*, scabies is considered a neglected tropical disease that mainly affects the young, elderly and the immunocompromised. The prevalence of scabies is highest in Western Pacific island nations. Despite the high prevalence and recent outbreaks in several high-income countries like the United Kingdom and New Zealand, scabies remains a frequently misdiagnosed disease. Scabies diagnosis often depends on clinical suspicion and the experience of the examining clinician. It is often misdiagnosed as other dermatological conditions like eczema or insect bites due to the low sensitivity of several clinical signs and a lack of a clinically available objective gold standard. This manuscript presents a comprehensive review of tests used to detect scabies, discusses the risks of misdiagnosis and offers suggestions to improve diagnosis. Three databases (PubMed, Scopus, and Web of Science) were searched to identify relevant articles for this review. Diagnostic methods reviewed in this paper include visual inspection, skin scraping, ink test, adhesive tape test, dermoscopy, videodermoscopy, and polymerase chain reaction (nested PCR). Molecular and immunological assays such as PCR and enzyme-linked immunosorbent assay (ELISA) have shown promising specificity and sensitivity of over 80%, which could be considered for diagnosing scabies, especially in cases where clinical signs are unclear. An objective laboratory test for diagnosing scabies is likely to improve control efforts. Use of the ‘clinical’ and ‘suspected’ categories of the International Alliance for Control of Scabies (IACS) criteria is also likely to lead to improved diagnosis and treatment of more cases, reducing the risk of misdiagnosis, progression to serious conditions, and limiting disease spread. A consensus on the standardized use of a sensitive objective diagnostic tool is urgently required for resource-poor communities.

## 1. Introduction

Scabies is a contagious disease caused by the ectoparasite *Sarcoptes scabiei* var. *hominis,* which is estimated to affect about 200 million people globally, as of 2023 [1]. The disease has a high prevalence among children, the elderly and the immunocompromised and disproportionately affects individuals from low-resource countries [2,3]. Typically, common scabies presents with a small, inflammatory, pruritic rash with crops of small lumps (papules) on the fingers, wrists, arms, legs and genitals, with itch that intensifies at night [4].

Increasingly, evidence indicates that scabies is not a benign condition, as it leads to several serious illnesses, some of which can be fatal. The intense itch from scabies often leads to skin abrasion and the secretion of proteins that impair innate immunity, making the host susceptible to secondary bacterial skin infection [5]. These infections may lead to further complications such as post-streptococcal glomerulonephritis [6,7], acute rheumatic fever (ARF) and rheumatic heart disease (RHD) [8,9,10].

An issue with scabies is misdiagnosis that results in underestimating the true prevalence of the disease, leading to poor treatment efficacy and disease spread. Due to its resemblance to other skin conditions like insect bites, dermatitis and eczema, it is often hard to accurately diagnose scabies [11,12,13]. In addition, some individuals may have an asymptomatic phase of their scabies infection, which is why treatment of asymptomatic household members of the index case is recommended.

The inconsistent and varied approaches to diagnosing scabies lack a sensitive and objective gold standard [4]. Scabies diagnosis may be considered on a spectrum of certainty rather than as a binary confirmation, with varying accuracy. Microscopic identification of burrows, mites, eggs or feces is highly specific when present, although the absence of these signs does not exclude the disease (poor sensitivity), which leads to ongoing spread of the mite. To improve scabies diagnosis, objective diagnostic tools could be enhanced, and a new gold standard could be established based on a method that demonstrates both diagnostic sensitivity and specificity.

In this study, we review existing methods for diagnosing scabies, assess their diagnostic accuracy, and consider emerging tests such as quantitative polymerase chain reaction (qPCR) to objectively diagnose the disease. This will include a detailed analysis of the diagnostic methods, their advantages and shortcomings and results from past studies that have used these respective methods. This will provide a basis for comparison of diagnostic accuracy for choosing an optimal method. As scabies misdiagnosis is a common issue, this section will also address the need to differentiate it from other skin conditions like eczema or insect bites and prioritize the accurate detection of the disease. Finally, the International Alliance for the Control of Scabies (IACS) criteria are critically reviewed to determine their role in accurate scabies diagnosis.

## 2. Materials and Methods

A literature search was conducted in PubMed, Scopus, and the Web of Science database through 31 December 2025. To narrow the search results, the inclusion criteria were restricted to human subjects only and to English-language studies. In general, the following Medical Subject Headings (MeSH) terms were used with each of the methods: “sensitivity”, “specificity”, “diagnostic accuracy” and/or “mass screening”. Thereafter, a focused search was conducted for different diagnostic methods using appropriate MeSH terms. This included: “visual inspection”, “skin scraping”, “microscopy”, “ink test”, “adhesive tape test”, “dermoscopy”, “videodermoscopy”, “dermatoscopy”, “videodermatoscopy”, “PCR”, “ELISA” and “IACS”. For the review on scabies and misdiagnosis, the following search terms were used: “scabies”, “eczema”, “dermatitis”, and “misdiagnosis”. The references of these selected studies were also reviewed to consider additional and relevant studies. The search strategy has been provided in Supplementary Table S1.

## 3. Scabies Misdiagnosis

Scabies diagnosis can be made by a combination of history and examination or using either invasive and non-invasive tools, each of which possesses its own set of advantages and limitations. Classic scabies patients often present with intense itching, which often worsens at night, due to symmetrically distributed erythematous papules, pustules or burrows found commonly in the interdigital spaces, volar wrists, waistline, umbilical region, axillae, buttocks and genital regions [14,15]. The presence of clear signs is relatively easy to diagnose with appropriate clinical knowledge, at least in low-resource regions with limited access to sophisticated techniques such as microscopy.

However, the diagnosis of scabies may be challenging due to a wide range of clinical signs, especially for patients with atypical manifestations and modification of clinical signs due to the frequent use of topical corticosteroids in response to a supposed diagnosis of eczema [16]. The traditional “gold standard” for diagnosis of scabies is based on visualization of mites, eggs, or feces on microscopy, high-powered imaging or dermoscopy [14,15,17] by an experienced clinician. Access to diagnostic techniques like microscopy is often limited, and its ability to confirm scabies is frequently hindered by low sensitivity [4] potentiating the spread of the mite and the emergence of further cases. A frequent complication of untreated scabies, such as impetigo, abscess, ulceration, or cellulitis, may further complicate the clinical picture, with the focus of diagnostic effort centred on the bacterial complications rather than scabies as the underlying cause. In addition, non-specific manifestations in the form of secondary eczematization and impetiginisation are often present in scabies patients [18]. The overlap of conditions can lead to misdiagnosis, especially in cases where typical burrows are missing, hard to detect, or obscured by the complications of scabies, as mentioned above. In clinical practice, dermatitis observed alongside scabies may represent an inflammatory secondary reaction to scabies, rather than an independent primary condition.

Incorrect diagnosis and treatment of scabies could lead to scabies incognito, a condition leading to unusual clinical presentation of scabies due to treatment with systemic or topical corticosteroids [19]. Topical or systemic corticosteroids reduce the production of inflammatory cytokines and inhibit cell-mediated immunity, thereby reducing inflammation and making typical papules and burrows less obvious [20]. Skin lesions observed in patients with scabies incognito are nonspecific and therefore harder to detect [19].

### 3.1. Overlap with Eczema or Dermatitis

Symptoms of scabies and dermatitis, such as itching, papules, and excoriation, often overlap. However, there are subtle differences. Scabies is contagious, causing symptoms among several household or institutional contacts [21] and is more likely to lead to intense itch, often worsening at night [22]. Dermatitis or eczema, on the other hand, is not contagious [23,24,25], has a chronic, relapsing pattern [26] and is often caused by irritants or allergens [27]. Diagnosis can be even more complicated in crusted scabies, as it is often misdiagnosed as psoriasis, irritant dermatitis, or eczema. Associated delays in effective treatment of crusted scabies significantly increase the difficulty in treating it once recognized [28].

Evidence of misdiagnosis comes from skin conditions initially labelled as other diseases that ultimately turn out to be scabies after follow-up. Such instances are also seen in high-income countries. A 2022 study reported 6 cases of misdiagnosis across several conditions, including nummular eczema, condylomata acuminata, drug-induced atopic dermatitis, contact dermatitis, and an autoimmune skin disorder, highlighting the need to exclude scabies before diagnosing an allergy [12]. Even in ideal situations, checking for specific signs of scabies can lead experts to miss scabies cases. Since lesions of eczema or atopic dermatitis can have clinical features similar to those of scabies, microscopic examination or other laboratory tests for scabies are warranted in such cases [12]. Scabies has also been seen to present as apparent relapsing atopic dermatitis, especially when clinicians are influenced by a history of dermatitis diagnosis [29].

Relying on specific clinical signs, such as burrows, mites (delta-wing signs), and their components, can lead to misdiagnosis. This was seen in a study where 23 patients with scabies in China were misdiagnosed as having eczema or dermatitis. None of the patients had burrows detected or secondary pyogenic infections in their lesion morphology [11]. Such cases are often mistreated with oral antihistamines and topical corticosteroids, leading to worsening of symptoms and delayed diagnosis.

Use of topical or systemic corticosteroids can modify the clinical presentation [20] and temporarily suppress itch and inflammation, while promoting mite growth. Improper use of high-potency steroids, including clobetasol and betamethasone, can result in severe complications like Cushing syndrome and other endocrine disorders [30]. In severely immunocompromised cases, misdiagnosis followed by use of corticosteroids for extended periods can allow scabies to progress to crusted or nodular subtypes, increasing the risk of bacterial sepsis and even death [31].

### 3.2. Effect of Population Group or Settings on Scabies Misdiagnosis

Scabies detection can be more challenging in certain populations. Among older adults, for example, scabies may be present in atypical forms, with complaints of itch being misunderstood for other conditions common in old age [32]. This may lead to outbreaks and subsequent high costs of controlling the disease in long-term care facilities [33]. The situation can be even more challenging in cases of dementia, where complaints of itch are not made, and pruritic marks are frequently absent, making the diagnosis difficult [34]. Misdiagnosis may result, leading to prescribing antifungal or steroid creams with no visible improvement and ongoing spread.

Under-recognition of skin conditions is also common in institutional [35] and resource-poor settings [5] due to a paucity of diagnostic testing facilities. This can be due to factors such as doctors’ inexperience, hospital overcrowding, and a high burden of skin infections in endemic settings, leading to normalization [36]. Delayed diagnosis can lead to the spread of the disease, especially in institutional settings. Such cases are common, as was seen in Taiwan, where forty-four episodes of delayed diagnosis of scabies were identified after chronic steroid use and long duration of hospitalization [37].

Misdiagnosis of scabies is not just restricted to low-resource countries, but is also highly prevalent in high-income countries like New Zealand, where the disease was common, and misdiagnosis was frequent in Auckland educational institutions [13]. Scabies misdiagnosis can be a challenge, especially due to a lack of awareness or experience, particularly in areas where it is not endemic. Further reasons for misdiagnosis might include failing to consider comprehensive patient histories, including symptoms such as itch or close contact with someone with itch, and failing to suspect the disease in a patient with apparently good hygiene who presents with atypical signs. In such cases, improved access to and use of diagnostic facilities, especially in institutional settings, is extremely important to reduce misdiagnosis rates and contain the risk of spread. Use of more sensitive objective tests such as qPCR, along with the deployment of emerging technologies such as artificial intelligence to analyze clinical images, might help improve diagnosis and prevent disease spread.

## 4. Diagnostic Methods

At present, several methods for the diagnosis of scabies exist. There are two main ways of diagnosing scabies: one seeks confirmation with visualization of mite material, which is specific but insensitive, whereas the other focuses on less specific but more sensitive signs with a greater assessment of the full clinical picture (e.g., IACS ‘clinical’ and ‘suspected’ categories). In many situations, the response to treatment (i.e., resolution of rash) can also retrospectively add further evidence for a suspected diagnosis. The more specific approach is often only accessible to specialists with specialized equipment, whereas the latter is more widely applicable.

One of the primary issues with diagnosis is the lack of a universally accepted gold standard [4]. When evaluating the diagnostic accuracy of different methods, the gold standard or reference standard often varies across studies, limiting the comparability of reported diagnostic accuracy estimates. Some studies may use microscopic identification of mites, eggs or scybala as the reference standard, whereas others may rely on clinical diagnosis, dermoscopy, the IACS diagnostic criteria, or a composite of several methods. As these reference standards differ in their sensitivity, specificity, and degree of diagnostic certainty, the reported sensitivity and specificity of the index tests are influenced by the method used to evaluate them. Since currently available diagnostic techniques are not sufficiently sensitive and specific across all clinical settings, scabies diagnosis often relies on a combination of clinical judgement and supportive laboratory findings rather than a single definitive test. The lack of a sensitive objective laboratory gold standard means that using a syndromic approach and divorcing diagnosis from a ‘decision to treat’ or a ‘trial of treatment’ is often pragmatically used in clinical practice, considering the ease of treatment and the lack of treatment-related side effects. Confusingly, some tests such as dermoscopy can be labelled as ‘sensitive’ when compared against another gold standard that is assessing similar biological evidence of disease status, such as from skin scraping. Lack of access to laboratory testing means a formal, confirmed diagnosis of scabies is frequently not made. Formal diagnosis of scabies, however, may be important, particularly when other diagnoses, such as insect bites or eczema and past treatment introduce further diagnostic uncertainty.

This section critically reviews the major diagnostic approaches, highlighting their strengths and limitations, and compares their sensitivity and specificity across different techniques. The diagnostic methods are divided into three sections: traditional, clinical signs and recent advancements.

### 4.1. Traditional Method: Visual Inspection

Confirmation of scabies in low-resource countries with high prevalence may be tough due to the paucity of resources [38], and diagnosis often relies solely on the presentation of characteristic symptoms and signs [39]. However, diagnosing scabies based on symptoms can be straightforward for trained clinicians and is often useful in community settings.

Traditionally, dermatologists assess the presence of burrows and visualize the mite head in situ (delta-wing sign), as well as the distribution of skin lesions, to diagnose scabies. Other methods used by non-specialists tend to focus on suspected cases with small papules and tiny raised or flattened burrows, with symptoms of itch, especially at night. The most common sites of scabies infestation that can be clinically examined for lesions are the hands, fingers, wrists and ankles, whereas papules without obvious infestations can be found in cases of mild disease burden [39]. These lesions represent an inflammatory and immune response of the body due to infestation by the scabies mites [40]. Precise ascertainment of scabies lesions is challenging, especially in the tropics, where papules and symptoms such as itching can mimic other diseases such as onchocerciasis [41]. To complicate matters, in cases of severe infections characterized by boils, the presence of female scabies mites is fewer than in counterparts with no infections [42,43]. Additionally, the presence of burrows or delta-wing signs can be difficult to discern without magnification or in pigmented skin [44].

Studies reporting the sensitivity or specificity of visual inspection are not available, although a few studies have attempted to estimate them under select conditions. One study, for example, aimed to use Community Health Workers (CHWs) to diagnose scabies based on skin lesions, lesion distribution, history of itch, and the presence of symptoms among close contacts [45]. When their assessment was compared to evaluations by trained physicians, the sensitivity and specificity of the CHWs were estimated at 85% and 100%, respectively.

Detection of scabies at the primary health care level, based on diffuse itching with observable lesions in at least two typical sites, along with the presence of a household member with itching, had a sensitivity of 100% and a specificity of 97% [46]. The test results were compared with the diagnosis made by a dermatologist, which was eventually confirmed using laboratory tests. In another similar study conducted in the Solomon Islands, four non-expert health workers were trained to diagnose scabies and impetigo, and the results were blinded and matched against the evaluations of two doctors, who were considered the ‘gold standard’ [47]. The sensitivity of diagnosing all scabies cases by the non-expert health workers against the gold standard was 55%, with a specificity of 89.9%, while the sensitivity of diagnosing only moderate and severe cases was up to 93.5% [47]. The low sensitivity for mild cases suggests a risk of underestimation of scabies cases through visual inspection by non-expert health care workers, who may see mild cases as a variant of normal skin. Variation in diagnostic accuracy of visual inspection by healthcare workers is likely to be due to the lack of an acceptable reference standard set by dermatologists to validate these tests. While this method can be used to detect moderate or severe cases in resource-poor areas with high prevalence, clinical diagnosis by experienced clinicians, followed by confirmation with objective tests, could improve diagnostic accuracy. However, combining diagnostic methods would be impractical in high-caseload areas.

### 4.2. Methods Relying on Specific Microscopic or Clinical Signs

Certain methods of detecting scabies rely heavily on specific clinical signs, the detection of which is enhanced by tools. These methods are useful in confirming scabies upon visualization of these signs. However, visualization is often restricted in the case of pigmented skin [48], or in cases where symptoms might have been altered by the improper use of corticosteroids [20,49]. These methods are also difficult to carry out in low-resource settings. Such methods include skin scraping, ink test, adhesive tape test, dermoscopy, and videodermoscopy.

### 4.3. Skin Scraping

Skin scraping is the most used technique for confirming scabies. This method involves a skin scraping obtained using a sterile blade or a microscopic slide, sometimes after applying mineral oil to the lesions [50,51]. Potassium hydroxide or saline is then added to the scrapings when the sample is placed on a slide [52]. The scrapings are then examined under a microscope to confirm the presence of scabies mites, eggs or fecal pellets [45,53]. These fecal pellets are dissolved using potassium hydroxide and are stained with Chlorazol Black E [53].

Although this method has high specificity [54,55], its sensitivity is quite low [44,50] due to various factors. These include the operator’s mounting technique, the low burden of the scabies mite and individual sampling [17,44]. This technique may be useful when used by experienced experts and thus requires extensive training. Other limitations of this method are the risk of introducing secondary bacterial infection and the time- and labour-intensive nature of the procedure involved [55,56]. Additionally, skin scrapings may be painful and poorly tolerated, especially in children [55,56].

Separate studies reporting the diagnostic criteria for skin scraping have shown a low sensitivity for this method. Studies comparing the diagnostic accuracy of skin scraping against clinicians’ assessment and other methods have reported a lower than 50% sensitivity for skin scraping [57,58,59].

### 4.4. Ink Test

The first reporting of the burrow ink test (BIT) dates back to 1981, when it was routinely used to diagnose scabies cases in a French hospital [60]. The method involved smearing a scabies lesion with ink using a fountain pen, then wiping off the excess ink with an alcohol-saturated gauze. A burrow made by a scabies mite, resembling a typical curvilinear structure, was traced with ink. This ‘zigzagged line’ can be seen with the naked eye. This is a simple and cost-effective test that can be used to visualize pathognomonic burrows [61]. Although this is a rapid point-of-care test suitable for use in low-resource regions, it has the drawback of being unable to distinguish old lesions from new ones [52]. This test is in sensitive due to a lack of adequate mites in many cases and cannot be used to rule out scabies [62]. Conversely, a negative test cannot confirm the absence of the disease, thus making it essential to carry out further tests like dermoscopy or microscopic examination for validation [61]. However, in patients with richly pigmented skin, typical dermoscopic features such as the delta-wing jet sign are absent, making detection of the mite challenging [63]. In one study, the BIT was helpful in diagnosing and treating a patient to recovery, whereas dermoscopic examination had failed to detect the disease [64]. Past studies have also recommended the use of superficial shave biopsy on scabies lesions to confirm the results of a BIT [60]. The probability of true false-positives from a BIT is, however, less, as other conditions do not mimic the characteristic burrow morphology [60].

### 4.5. Adhesive Tape Test

This method uses adhesive tape applied to suspected burrow sites, followed by rapid removal and microscopic examination for mites. Pulling off the tape extracts the stratum corneum along with the scabies mites and their products [50]. The tape, attached to a slide, can be stored at temperatures between 10 °C and 14 °C until the slides are examined [57]. This process enables visualization of the mite, feces, or eggs attached to the tape. The simplicity of the test, along with its high positive and negative predictive value, makes it a useful screening test [65]. In this test, the mites stuck to the adhesive test are unable to migrate, and there is less cell debris than in a slide prepared from cell scrapings [57]. This makes it a handy tool, especially for diagnosing scabies in resource-poor settings.

A study involving 113 participants investigating the diagnostic accuracy of different tests found that the sensitivity and specificity of the test were 68% (95% CI, 52–81%) and 100% (95% CI, 94–100%), respectively [57], compared to a gold standard of positive findings in any of dermoscopy, tape test and skin scraping. The study also found that the sensitivity of the adhesive test was unaffected by disease severity. In another study that assessed 100 patients using skin scraping, adhesive tape and dermoscopy, the adhesive tape was found to be the most sensitive of the three [59]. The study had used a clinician’s diagnosis as the gold standard. The sensitivity of the adhesive test in this study was 69.6%, with a specificity of 100%. In a study by Katsumata & Katsumata (2006), adhesive tape was used to detect scabies among thirty-one bedridden elderly patients in a hospital [66]. This method was deemed particularly useful for elderly patients with thin, dry skin. Despite its high specificity and other advantages, its sensitivity is low [59,66], meaning that it is often used with other tests, such as dermoscopy. This method, combined with fluorescence staining, is believed to improve the diagnostic accuracy of scabies detection and is less time-consuming. This is confirmed by a study that used dermoscopy to visualize a sinuous burrow in the subject’s finger webs and then validated it with transparent adhesive tape, which revealed a hatching mite egg [67].

### 4.6. Dermoscopy

Dermoscopy involves visualization of scabies burrows using microscopic magnification. Even a low-magnification pocket-held dermoscope with 10× magnification is a useful, specific tool for detecting scabies [68]. As a non-invasive diagnostic technique, it is painless, improves patient compliance, and is especially useful in situations where obtaining skin scrapings from certain patients is challenging. This technique detects the delta-wing jet pattern in the skin to determine whether the mite is present. The delta jet wing pattern, also referred to as the delta glider or jet with contrail, is indicative of scabies burrows and scabies mites [17]. While the burrows appear as white, structureless lines, the head and anterior legs of the mite are represented by a dark brown, triangular structure, with eggs appearing as small ovoid structures inside the burrows [17]. Although it sometimes poorly visualizes eggs and feces, with proper training, it is possible to identify the delta-wing sign and confirm the presence of the disease [50]. Accurate identification of mites and eggs is possible at magnifications ranging from 40× to 1000×. This diagnostic method, however, is relatively more expensive than the other alternatives. Dermoscopy can also be time-consuming in some cases. While examining individual lesions by an experienced clinician can be quick, investigating multiple or atypical lesions can take considerably longer.

The diagnostic accuracy of dermoscopy has been evaluated in several studies to date. A prospective, nonrandomized, evaluator-blinded study involving 756 patients comparing the diagnostic accuracy of different methods found that the sensitivity of dermoscopy was high at 91% (95% CI, 86–96%) [68]. However, the specificity of dermoscopy was lower (86%; 95% CI, 80–92%) compared to the other methods, such as microscopic examination of skin scrapings, which is itself a low-sensitivity method. The study highlighted the method’s high sensitivity, even for inexperienced dermoscopists, and emphasized that their accuracy improved over time [68]. In another study involving 113 patients, the sensitivity of dermoscopy (83%; 95% CI, 70–94%) was significantly higher than that of other tests, such as skin scraping (46%; 95% CI, 31–62%) and the adhesive tape test (68%; 95% CI, 52–81%) [57]. The method’s specificity was low in this study, with confounding caused by debris such as point bleeding, crusts, or dust particles that resembled the mite. Apart from these factors, pigmented skin can make the delta-wing sign difficult to visualize on dermoscopy. Finally, in a prospective study of 136 patients, skin scraping combined with dermoscopy was found to have higher diagnostic accuracy and was much more effective for diagnosing scabies [69].

### 4.7. Videodermoscopy

This is an improved version of dermoscopy that uses a video camera, optical fibres, and lenses connected to a computer, enabling up to ×1000 magnification [70]. Apart from visualizing the burrows at magnifications up to 100×, this method is highly useful for visualizing the mites, eggs, or feces at magnifications as high as 600× [56]. The ability to inspect the superficial dermis enables in vivo identification of burrows and scabies [71]. The entire process of assessing the skin surface through videodermoscopy takes about 3 to 5 min, making it a non-invasive, high-compliance test, particularly helpful in situations needing repeated testing to obtain a conclusive result [70]. This method has been observed to be preferred by patients as a post-therapeutic diagnostic technique over traditional methods to confirm the possible presence of scabies mites [70].

The high sensitivity and specificity of the videodermoscope have been confirmed in a limited number of studies. A study published in 2000 confirmed the method’s high specificity and a low probability of false-positive results by directly recognizing the scabies mite in vivo [72]. The paper also emphasized the reduced risk of accidental infection from blood-transmissible agents like human immunodeficiency virus (HIV) or hepatitis C virus (HCV) when using this method. A more recent study published in 2008 on high magnification videodermoscopy concluded that the test was accurate in detecting scabies, particularly for cases with non-specific clinical features, as it allows for clear detection of mites, eggs, or feces, which are generally not detected by traditional tests [71].

Dermoscopy, videodermoscopy, and microscopy rely on the direct visualization of highly specific signs such as mites or characteristic features such as burrows, eggs, or scybala. Their sensitivity is generally considered to be low for the majority of common scabies, where mite burden is low and pathognomonic lesions such as these are often difficult to identify. It is notable that the gold standard comparison test by which these tests are evaluated is usually one or a combination of tests that look for similar very specific signs. The limitations of all of these methods (being specific but not sensitive) suggest that other specific and sensitive objective laboratory methods of making a definitive diagnosis of scabies should be sought.

### 4.8. Molecular Tests

Advances in molecular testing in recent years have enabled high sensitivity and quantification of parasite load. Polymerase chain reaction (nested PCR) based on the cox1 gene of *S. scabiei* is an extremely sensitive test for diagnosing scabies [73]. DNA extracted from *S. scabiei* swab specimens and skin biopsies can be used to obtain accurate test results [55]. It is a useful diagnostic option in areas with large scabies cluster outbreaks, in which the response may be resource intensive. In a unique 2021 study, skin flakes from bed linen were used to detect scabies in asymptomatic patients without primary lesions [74]. The PCR test detected two additional cases beyond those detected by other tests, indicating promising potential for further use. In a study conducted in Seoul, where diagnoses made using IACS criteria were considered the gold standard, PCR was performed on 47 participants, yielding a sensitivity of 86% for confirmed scabies cases and a specificity of 100% (95% CI: 80–100%) [75]. In a recent development, nested real-time quantitative PCR (nRT-qPCR) with skin scraping was found to have a significantly higher sensitivity of 92.7%, exceeding that of all other tests [76]. As PCR depends on the physical presence of the mite or its residues, it can lead to low sensitivity when mite burden is low.

PCR tests are, however, more expensive than traditional tests like microscopy. In a study by Wong et al. from Hong Kong (2015), each test conducted using PCR and qPCR assays cost approximately $6.70 and $11.0, respectively, while the cost of a test using microscopy was $3.90 [16]. The authors explicitly noted that PCR assays were more expensive than microscopy and argued that the additional cost might nevertheless be justified in some settings since PCR could identify microscopy-negative cases, resulting in higher sensitivity [16]. The financial barrier is not just limited to the reagent cost per assay, as the PCR technique is also dependent on laboratory infrastructure. Thus, PCR is not considered a point-of-care test due to the high skill level required for implementation and the capital costs of laboratory equipment [77]. These considerations are particularly relevant in low-resource scabies-endemic communities where a diagnostic test may need to be deployed at community scale.

### 4.9. Serological and Immunological Assays

Serological and other immunological assays have also been investigated as alternatives to direct detection of the scabies mite. In an experimental animal study, an indirect ELISA using recombinant *Sarcoptes scabiei* cofilin protein as the antigen showed a reported sensitivity of 83.3% and specificity of 87.9% when evaluated using sera from *S. scabiei-infected* naïve rabbits [78]. Although these findings suggest potential diagnostic utility, validation in humans is necessary. Owing to its non-traumatic diagnostic technique, this method of diagnosis is repeatable, which increases the sensitivity of cluster scabies diagnosis [79].

In one of the earlier studies, ELISA using antigens derived from fox mites was evaluated to detect serum IgG in 41 patients with confirmed human scabies [80]. Although specific IgG was significantly higher in patients with scabies compared to healthy controls, the sensitivity was only 48% [80]. In another study by Walton et al., antibody responses to four recombinant mite proteins (CO8, FO4, GO3 and Ssag1.2) were examined using ELISA with secondary antibodies directed against IgA, IgG, IgG1, IgG4 and IgE [81]. In particular, an IgE ELISA directed against Ssag1.2 demonstrated a sensitivity of 88% and specificity of 100%, while an assay based on CO8 demonstrated a sensitivity of 76% and specificity of 100% [81]. Conversely, a study that tested sera of 91 patients for Ig, IgE, IgD, IgG and IgM antibodies to *S. scabiei* as well as to house-dust mites found that only 45%, 27% and 2.2% of the patients had measurable amounts of mixed Ig, IgG and IgE respectively that recognized scabies mite antigens [82]. Owing to the extensive antibody reactivity observed against house-dust mites, the study concluded that this cross-reactivity represents an important barrier to the development of serological assays based on crude mite antigens [82].

Serological assays conducted with laboratory diagnosis of scabies using whole-mite extracts are time-consuming and extremely expensive. This is due to the fact that the method relies on a source of antigen which cannot be routinely propagated in vitro, adding substantial logistic and production costs [52].

In addition to ELISA, dissociation-enhanced lanthanide fluorescent immunoassay (DELFIA) has been investigated for scabies diagnosis. In a study that measured IgE directed against the recombinant major scabies allergen Sar s 14.3 (rSar s 14.3) in 140 plasma samples from scabies-infested and control participants, a sensitivity of 100% and specificity of 93.75% was observed [83]. Such a finding is very promising; however, the test has not had wide uptake in clinical settings.

Since diagnosis using immunological assays does not depend on directly recovering the mite, eggs or scybala from an individual lesion, immunological assays are useful in patients with low mite burdens or lesions that are difficult to sample.

## 5. The IACS Criteria: A Much-Needed Improvement

The IACS criteria were launched in 2012 with an aim of recognizing the health impacts of the disease and prioritizing strategic policies to reduce its burden through the development of robust and sensitive diagnostic tests, leading to faster treatments [84]. After the WHO recognized scabies as a neglected tropical disease (NTD) in 2017, the need to integrate it into population-based surveys for other NTDs and to estimate its prevalence across regions became apparent. A four-round Delphi study involving an international panel of highly experienced clinicians was conducted to devise and adopt a new set of standardized criteria for the diagnosis of scabies [85]. This formalized the recognition of crops of small inflammatory papules (also, less commonly, vesicles, nodules, and pustules), in a distinct anatomical distribution, with supporting symptoms, as constituting a diagnosis of scabies—albeit with less certainty. This formalized the experience of clinicians working in high-prevalence areas, where symptoms of scabies and its complications were prevalent, but microscopic signs were infrequently found. A set of criteria was developed based on clinical symptoms, such as visualization of mites or their residue using a microscope or dermoscope (confirmed scabies—based on dermoscopic or microscopic signs) or visualization of burrows or ‘typical lesions’ (crops of inflammatory papules) in particular areas with typical distribution (clinical scabies) or typical or atypical lesions along with history features (itch or close contact with individuals with itch).

Another Delphi study was conducted in 2020, leading to the 2020 IACS criteria for diagnosing scabies. The intention was to standardize the diagnosis for communication and comparison and bring about homogeneity in epidemiological findings. This criterion also had three levels representing the degree of diagnostic certainty—confirmed scabies (level A), involving direct visualization of the mite or its residuals; clinical scabies (level B); and suspected scabies (level C), relying on assessment of less specific signs and symptoms [4].

The committee recommended extensive training for examiners using images published with the criteria for diagnosing scabies, and included training on other NTDs and skin conditions that often coexist with scabies [4]. The high sensitivity of this approach could lead to its adoption as a practical alternative in large-scale community studies with low resources. Many clinicians are still unaware of the updated IACS criteria, which include ‘clinical’ and ‘suspected’ cases of scabies, thereby increasing case detection. A global adoption of the IACS criteria is likely to improve diagnosis and lead to better treatment of scabies in the future.

Since then, a few studies have examined the accuracy of the IACS criteria for diagnosing scabies. A study published in 2020, conducted in Liberia, found that non-expert examiners could diagnose a large number of scabies cases with only two days of training [86]. The sensitivity of the IACS criteria for non-expert examiners, compared with the gold standard of clinical and dermoscopic examinations conducted by two expert examiners, was 85.4% (95% CI, 73–93%), with a specificity of 71.4% (95% CI, 56–96%).

The Liberian study also demonstrated the contrasting relative frequency of clinical signs of scabies. The study highlights the need to expand the definition of scabies to more sensitive signs, rather than those which are more specific, such as burrows or those indicating the presence of mites (‘delta wing’ and ‘wake’ signs). Of the 45 identified scabies cases, almost all (44; 97.8%) had papules, whereas less than half had burrows present (21; 46.7%). A small minority were positive by dermoscopy (6; 13.3%). This means that only one in every 7.5 scabies cases would be identified, and the use of dermoscopy as a sole means of diagnosis is unlikely to contain the ongoing spread of scabies.

This finding also supports the use of non-experts with minimal training for diagnosing scabies in low-resource areas. Thereafter, several studies, particularly in low-resource countries, have adopted the IACS criteria to estimate scabies prevalence [87,88,89,90,91]. One study specifically aimed to investigate the accuracy of the simplified IACS criteria [89]. Although no significant difference was observed in prevalence rates, the simplified criteria had a sensitivity of 82.3% (95% CI, 80.2%–84.2%) compared with the full IACS criteria.

## 6. A Comparison of the Diagnostic Methods

Each diagnostic method considered in this review has a unique set of advantages, limitations and diagnostic accuracy. Although available evidence of the diagnostic performance of these tests is limited and heterogeneous, and different studies use different reference standards, an attempt has been made to provide an overview of the advantages, limitations, and reported diagnostic accuracy of each method. As estimates of diagnostic accuracy were derived using non-standardized reference standards, comparisons across methods should be interpreted with caution. Table 1 provides a comprehensive summary of each method, while Table 2 presents a direct comparison based on several criteria.

## 7. The Way Forward

### 7.1. Improvements in Diagnostic Strategies

Traditionally, scabies diagnosis is centred on highly specific rather than sensitive findings, which inevitably lead to poor disease control and underestimation of prevalence. The IACS ‘clinical and suspected’ categories represent a shift toward more accessible and sensitive clinical signs and symptoms that are likely to improve disease control. While in most obvious and typical cases, scabies can be easily diagnosed through these clinical signs, it is important to realize that the disease can mimic other conditions and present a range of clinical symptoms. This makes accurate diagnoses difficult.

As this review shows, each diagnostic technique has shortcomings, leading to the absence of an objective and accessible gold standard. At present, there is no universal objective gold standard, leading to heterogeneity in prevalence estimates due to the use of different tests across settings. This leads to limitations on inference drawn from epidemiological findings.

Most methods, such as microscopy or dermoscopy, require expert training and access to resources such as laboratory facilities, which may not be feasible in low-resource countries with high scabies prevalence. Specific microscopic signs such as burrows, delta wings, eggs, or mite feces can be missed in cases of pigmented skin or prior steroid use (scabies incognito). Hence, owing to low sensitivity, if these criteria are used to guide clinical care, they will result in many false-negative cases, resulting in inadequate treatment and community spread of the disease.

Given the low sensitivity, treating scabies based on suspected IACS symptoms or treating close contacts despite the absence of obvious symptoms, as is currently recommended, is likely to be less of a public health problem than underdiagnosis. Since the side effects of scabies treatment are few, it is generally considered safe; the cost is low, and the potential benefit is high; the benefits most probably outweigh the risks for all but the lowest-risk patients.

### 7.2. Improvements in Treatment Strategies to Offset Low Sensitivity in Resource-Poor Settings

The current guidelines recommend a low threshold of 10% prevalence for mass drug administration (MDA) [92]. Considering the uncertainty around scabies diagnosis, prevalence in a low-resource community is often severely underestimated. Hence, rather than relying on tests that may lack sufficient sensitivity to establish the required prevalence threshold, MDA should be considered in resource-poor communities where the burden of symptoms is generally high among residents.

However, the risk of overtreatment leading to drug resistance [93] should not be ignored. In cases of treatment failure or reinfection, where diagnostic uncertainty persists, PCR could be particularly useful, as it can distinguish active infestation from residual infection and help avoid unnecessary retreatment.

To increase the rate of scabies detection, more sensitive clinical signs like crops of papules associated with itch and accompanying symptoms in the family, should be considered for scabies diagnosis as defined by the IACS ‘clinical’ and ‘suspected’ categories (Figure 1). Although these criteria have not yet been uniformly adopted by clinicians, widespread clinician-level uptake will facilitate improved control of scabies, since more specific signs are rarer. As the IACS criteria are relatively easy to apply, particularly in resource-limited settings, they should be considered the first-line approach for scabies diagnosis. Where diagnostic uncertainty remains after applying the IACS criteria, additional diagnostic tests may be considered based on the availability of resources within the country or setting (Figure 1). Considering the pros and cons of the various diagnostic approaches to scabies, we suggest that the IACS criteria be most suitably recognized as the ‘gold standard’.

Often, limited data on local scabies prevalence is available to guide a pre-test probability assessment of the likelihood of scabies. Hence, scabies surveys should be prioritized to enable decision-making for a community-wide control, with higher community prevalence justifying a lower threshold for initiating treatment. In resource-poor communities with a high prevalence of scabies and inadequate provision of MDA, a trial of treatment could be considered for anyone with itch, for example.

Objective molecular tests and immunological assays, such as PCR or ELISA, have adequate sensitivity and specificity but are unlikely to be implemented in low-income contexts where much of the disease burden exists. Nevertheless, improving surveillance and the accuracy of scabies diagnosis, even in higher-income contexts, is likely to be worthwhile and cost-effective, and to improve scabies control.

### 7.3. Decision to Treat: Dealing with Diagnostic Uncertainty

For effective control of scabies, treatment should be synchronized with the entire household, even in the absence of symptoms. Treatment policies, such as those recommending and demonstrating efficacy for mass population treatment (of the whole community, regardless of signs or symptoms) in a setting of prevalence greater than 10% [92], suggest that clinical diagnosis, or at least the decision to treat or not, should be tailored to the knowledge of the prevalence in the population of interest, which assesses pretest probability. This is important, since the reversible nature of the disease and the low frequency of side effects from treatments mean a low threshold for a therapeutic trial of treatment in cases of diagnostic uncertainty may be justified. The threshold to treat may be adjusted to the background prevalence of scabies in the region.

Because it is possible to miss potential cases using up-to-date clinical criteria during mass drug administration in endemic regions, studies have shown the superiority of treating entire populations over usual strategies of treating cases and contacts only. Ivermectin-based mass drug administration on one island led to a 67% (95% CI, 52–83) decline in scabies prevalence one year after treatment began, compared to 32% (95% CI, 14–50) in the island following standard care protocols [94]. This suggests that standard case detection and treatment of contacts have limitations for disease control.

Surveys using rigorous clinical definitions (e.g., IACS clinical and suspected) can help tailor guidelines for the ‘decision to treat’ scabies at a population level. This is particularly important in high-prevalence settings, where diagnostic tests often have low sensitivity, symptoms may be delayed, and health-care resources are limited. In such settings, the decision to treat may need to be informed not only by individual clinical findings but also by the local prevalence and severity of the outbreak. By contrast, diagnosis of an individual case remains primarily a test- or clinically based assessment. During outbreaks, however, treating only confirmed cases risks missing asymptomatic infections and untreated household contacts, allowing transmission to persist. Consequently, incorporating local prevalence data into treatment guidelines may improve the control of scabies, particularly in low-resource settings. This underscores the value of local prevalence surveys for informing context-specific approaches to scabies diagnosis and treatment.

## 8. Conclusions

The similarity of scabies to other skin conditions has major implications, especially among the elderly, the immunocompromised, and in low-resource settings. The standard diagnostic methods, focusing on infrequent signs, are not sufficiently sensitive and miss many cases, leaving them undiagnosed. Lack of clinically accessible, sensitive and objective tests has a detrimental impact on scabies control and prevalence estimation. Global adoption of more sensitive emerging diagnostic criteria such as the IACS could lead to more accurate diagnosis and treatment for a larger number of patients, especially those with atypical symptoms. Local surveys may also play a role in tailoring treatment guidelines. Synchronizing treatment through policies like MDA could be considered in endemic resource-poor communities where accurate diagnosis of scabies is challenging.

## Figures and Tables

**Figure 1:**
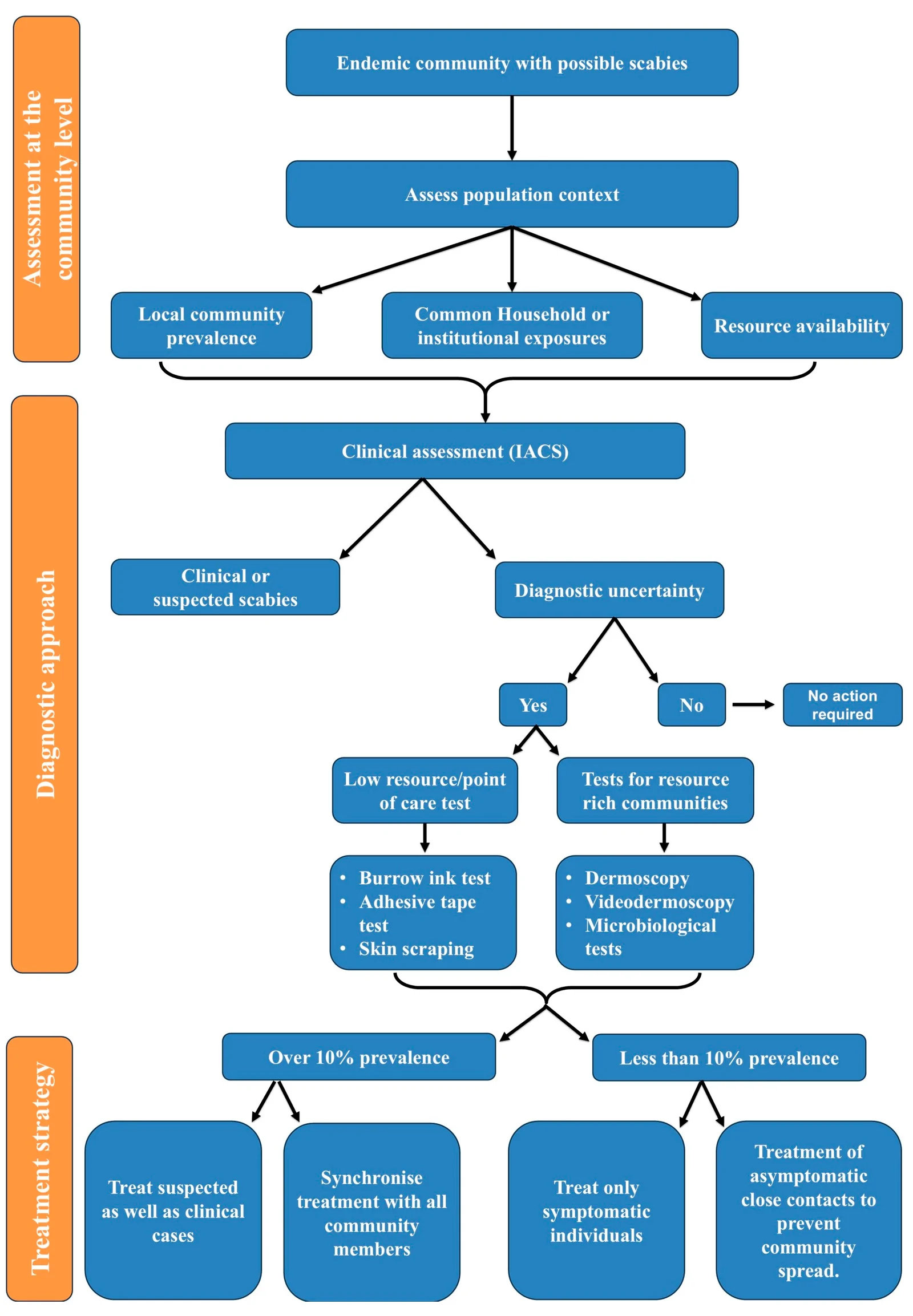
Proposed framework for effective diagnosis and treatment of scabies.

**Table 1:** Summary of diagnostic methods used to diagnose scabies.

Diagnostic Methods/Criteria	Technique Used	Advantages	Disadvantages	Sensitivity Reported from Past Studies *	Specificity Reported from Past Studies *
Visual inspection	Clinical assessment of scabies signs, including assessment of burrows, lesions and history of itch.	Easy, cost-effective and useful for community-wide screening in resource-poor settings.	Dependent on the experience and expertise of clinicians. Poor detection of mild cases, especially in cases of eczematization or impetiginisation. No universal reference standard.	55–85%	89.9–100%
Skin scraping	Identification of mite particles, including eggs or fecal pellets, under a microscope.	Confirms infestation where mite particles are visible.	Sometimes painful, hence not well tolerated by children. Labour-intensive and requires expertise from the operator.	<50%	High
Burrow ink test	Burrow made by scabies mite traced using ink smeared from a fountain pen.	Cost-effective, serves well as a rapid point-of-care test, useful in resource-poor settings.	Unable to distinguish old burrows from the new ones. A negative test cannot confirm the absence of scabies. Ineffective for patients with richly pigmented skin.	Not consistently reported	High (few false positives)
Adhesive tape test	Use of adhesive tape to extract the stratum corneum along with scabies mites and their products.	Sensitivity is unaffected by the severity of the disease. Useful particularly for elderly patients with thin and dry skin. Simple, painless and cost-effective.	Usually requires confirmation.	Around 70%	100%
Dermoscopy	Magnified visualization of the delta-wing sign.	Non-invasive and painless.	Prohibitively expensive; requires training of the operator.	85–90%	>85%
Videodermoscopy	High-magnification digital dermoscopy.	Most accurate visualization of mites, eggs and feces. Non-invasive, requires less time.	High cost, limited availability.	High	High
Molecular tests	Detection of *S. scabiei* DNA.	Useful for minimal asymptomatic infection.	Expensive, requires laboratory facilities.	>85%	>100%
Serological and immunological assays	Detection of host antibodies against *Sarcoptes scabiei* antigens. Assays may use crude/whole-mite extracts or recombinant mite antigens and detect antibodies such as IgG or IgE.	Non-invasive/non-traumatic and repeatable; does not require direct demonstration of mites, eggs or scybala. Useful in patients with low mite burdens.	Diagnostic performance varies substantially according to antigen and antibody detected; cross-reactivity with house-dust mites may reduce specificity.	76–88%—recombinant-antigen ELISAs (human study); 100%—rSar s 14.3 DELFIA (human study);	100%—recombinant-antigen ELISAs (human study); 93.75%—rSar s 14.3 DELFIA (human study)
IACS ^$^	Standardized clinical diagnostic criteria of scabies based on clinical signs and history.	Useful for large-scale community studies with low resources, homogeneous in epidemiological findings, minimal training required.	Lower specificity.	>80%	>70%

* The estimated sensitivity and specificity of each diagnostic method may be biased when evaluated against a reference standard that itself has limited sensitivity. Scabies diagnosis currently lacks a universally accepted gold standard. A global adoption of an objective and reliable reference standard is therefore needed to enable valid comparisons of diagnostic accuracy across different diagnostic methods. ^$^ The IACS should be considered a diagnostic criterion rather than a diagnostic test. Since it is suitable for use in community settings and has demonstrated relatively higher sensitivity than several other diagnostic approaches, it has been included in the table for completeness and practical evidence.

**Table 2:** Comparative analysis of diagnostic methods used for scabies diagnosis.

Diagnostic Methods/Criteria	Sensitivity	Specificity	Cost	Expertise	Invasive	Point-of-Care	Suitable for Low-Resource Settings
Visual inspection	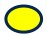	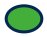	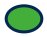	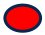	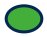	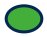	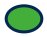
Skin scraping	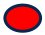	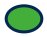	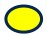	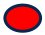	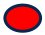	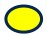	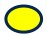
Burrow ink test	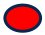	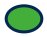	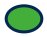	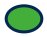	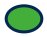	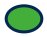	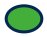
Adhesive tape test	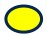	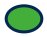	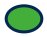	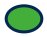	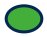	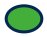	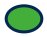
Dermoscopy	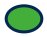	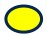	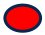	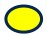	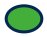	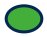	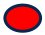
Videodermoscopy	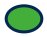	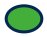	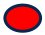	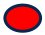	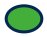	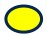	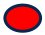
Molecular tests	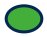	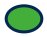	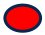	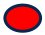	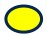	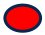	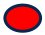
Serological and immunological assays	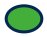	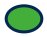	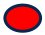	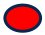	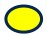	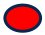	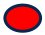
IACS	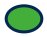	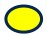	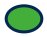	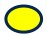	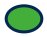	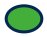	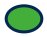

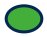
 = favourable; 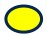 = moderate; 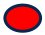 = less favourable; the visual inspection method looks for specific signs of scabies, such as the presence of papules in high-risk areas, typical lesions, presence of itch, etc. Other methods like skin scraping and dermoscopy rely on visualization of the mite for confirmation. The IACS criteria, on the other hand, also account for ‘suspected scabies’, which looks for additional evidence like contact history with a person with itch. Including ‘suspected scabies’ would increase sensitivity and enable synchronized treatment across households as well.

## Data Availability

No new data were created or analyzed in this study. Data sharing is not applicable to this article.

## Supplementary Materials

The following supporting information can be downloaded at: https://www.mdpi.com/article/10.3390/jcm15197346/s1, Table S1: Search strategy.
