# Supplementary material for: Scabies Diagnosis: A Narrative Review of Diagnostic Methods, Clinical Misdiagnosis, and Emerging Directions
Source: J Clin Med. 2026 Sep 22;15(19):7346. doi: 10.3390/jcm15197346 (PMC13666215; doi:10.3390/jcm15197346)
Supplement: Supplementary file 1 [file jcm-15-07346-s001.zip › jcm-4555652-supplementary.pdf]

Table S1: Search strategy

PubMed Search: 10/09/2025

Search	Query	Results
PubMed		
#1	Scabies	6,142
#2 Diagnostic Methods	((((((((((visual inspection) OR (skin scraping)) OR (microscopy)) OR (ink test)) OR (adhesive tape test)) OR (dermoscopy)) OR (videodermoscopy)) OR (dermatoscopy)) OR (videodermatoscopy)) OR (PCR)) OR (ELISA)) OR (IACS)	2,065,172
#3 Diagnostic accuracy	((((((sensitivity) OR (specificity)) OR (diagnostic accuracy)) OR (mass screening)))	7,143,043
#4	#2 AND #3	611,150
#5	#1 AND #4	199
#6	#5 [Restricted to Humans and English language]	76

Scopus

Search	Query	Results
#1	Scabies	10,419
#2 Diagnostic Methods	((((((((((visual inspection) OR (skin scraping)) OR (microscopy)) OR (ink test)) OR (adhesive tape test)) OR (dermoscopy)) OR (videodermoscopy)) OR (dermatoscopy)) OR (videodermatoscopy)) OR (PCR)) OR (ELISA)) OR (IACS)	4,002,991
#3 Diagnostic accuracy	((((((sensitivity) OR (specificity)) OR (diagnostic accuracy)) OR (mass screening)))	4,768,436
#4	#2 AND #3	384,634
#5	#1 AND #4	218
#6	#5 [Restricted to Humans and English language]	124

Web of Science

#1	Scabies	2,650
#2 Diagnostic Methods	((((((((((visual inspection) OR (skin scraping)) OR (microscopy)) OR (ink test)) OR (adhesive tape test)) OR (dermoscopy)) OR (videodermoscopy)) OR (dermatoscopy)) OR (videodermatoscopy)) OR (PCR)) OR (ELISA)) OR (IACS)	2,032,782
#3 Diagnostic Methods	((((((sensitivity) OR (specificity)) OR (diagnostic accuracy)) OR (mass screening)))	2,098,642

**#4**	**#2 AND #3**	**143,572**
**#5**	**#1 AND #4**	**61**
**#6**	**#5 [Restricted to Humans and English language]**	**58**
